# Risk of postpolypectomy bleeding in patients taking direct oral anticoagulants or clopidogrel

**DOI:** 10.1038/s41598-021-82251-y

**Published:** 2021-01-29

**Authors:** Gwang-Un Kim, Sinwon Lee, Jaewon Choe, Sung Wook Hwang, Sang Hyoung Park, Byong Duk Ye, Jeong-Sik Byeon, Seung-Jae Myung, Suk-Kyun Yang, Dong-Hoon Yang

**Affiliations:** 1 Health Screening and Promotion Center, Asan Medical Center, University of Ulsan College of Medicine, Seoul, Korea; 2 Department of Gastroenterology, Asan Medical Center, University of Ulsan College of Medicine, Seoul, Korea

**Keywords:** Colonoscopy, Gastrointestinal diseases

## Abstract

The usage of direct oral anticoagulants (DOACs) to prevent and treat thromboembolic events is gradually increasing. We aimed to evaluate the outcomes of patients taking DOACs after polypectomy. We retrospectively reviewed 131 patients taking DOACs and 270 taking clopidogrel who underwent polypectomy between November 2010 and December 2017. The risk of delayed postpolypectomy bleeding (PPB) was evaluated and compared. A total of 989 polyps were removed (320 polyps in the DOAC and 669 polyps in the clopidogrel group). DOACs and clopidogrel were discontinued for 2.8 ± 1.7 days and 5.8 ± 2.5 days before polypectomy, respectively. DOACs and clopidogrel were restarted on 1.6 ± 2.9 days and 1.7 ± 1.1 days after polypectomy, respectively. According to per polyp analysis, delayed PPB rate was 1.6% in both groups (*p* = 0.924). Logistic regression analysis was performed after propensity score matching and revealed that DOACs did not increase the delayed PPB risk compared to clopidogrel (OR 0.929, 95% CI 0.436–1.975, *p* = 0.847). With the majority following the antithrombotic discontinuation guidelines, the incidence of delayed PPB was 3.1% in the patients taking DOACs. The delayed PPB risk was not greater in those taking DOACs than in those taking clopidogrel.

## Introduction

Colonoscopic polypectomy is effective in preventing colorectal cancer (CRC) and reducing CRC-related mortality^1^ but carries a risk of procedure-related adverse events. Hemorrhage is one of the significant complications related to polypectomy. Patients taking antithrombotic medication are exposed to a greater risk of delayed postpolypectomy bleeding (PPB) than those not taking antithrombotic agents^2,3^. Current guidelines suggest discontinuation of antiplatelets and anticoagulants according to the compromise between the bleeding and thromboembolic risk considering endoscopic procedures, category of antithrombotic agents, and the medical condition requiring anticoagulation^4,5^. For example, clopidogrel increases the risk of PPB^3,6^, but aspirin does not^3^. In addition, discontinuing both clopidogrel and aspirin in patients on dual antiplatelet therapy for drug-eluting coronary stents increases the risk of early-onset stent thrombosis compared with stopping clopidogrel only^7^. The current guidelines recommend maintaining aspirin and stop clopidogrel 5–7 days before polypectomy^8^. Therefore, understanding the risk of bleeding with each drug is essential to determine the antithrombotic agent cessation strategy in relation to polypectomies.

Compared with warfarin, a representative vitamin K antagonist, direct oral anticoagulants (DOACs) take advantage of a rapid onset, little drug interaction, and no need for regular monitoring of anticoagulation effect. Thus, DOACs are increasingly selected for patients with nonvalvular atrial fibrillation (AF), venous thromboembolism, and other hypercoagulable conditions^9–11^. According to meta-analyses, the major gastrointestinal (GI) bleeding risk is lower in patients with DOACs than in those with vitamin K antagonists^12–14^. However, there is still a lack of evidence regarding the risk of PPB in those taking DOACs. Interestingly, a recent large-scale retrospective study about postpolypectomy adverse events suggested that not only warfarin but also clopidogrel carried a higher risk of PPB than DOACs^15^. Because clopidogrel has been used for a long time, it is familiar to the clinician and the associated risk of PPB has been well-investigated through previous studies. Therefore, the risk of PPB in those taking clopidogrel would be a good reference to assess the risk of PPB related to DOACs. Here, we aimed to investigate the adverse events related to polypectomy in patients taking DOACs or clopidogrel and to assess the risk factors of delayed PPB.

## Results

### Characteristics of the patients

The average age was 71.7 ± 9.01 years in the DOAC and 67.5 ± 9.34 years in the clopidogrel group (*p* < 0.001). In the DOAC group, the number of patients taking each drug was 65 for rivaroxaban, 33 for apixaban, 9 for edoxaban, and 24 for dabigatran. Seven patients in the DOAC group (4 for rivaroxaban, 2 for apixaban, and 1 for edoxaban) had impaired renal function. The average duration of antithrombotic discontinuation before the procedure was 2.82 ± 1.74 days for the DOAC and 5.84 ± 2.25 days for the clopidogrel group. Nineteen patients in the DOAC and 54 in the clopidogrel group discontinued the respective drug before polypectomy for a shorter duration than that suggested by the guidelines; thus, the antithrombotic guideline compliance rate was 85.5% in the DOAC and 80.0% in the clopidogrel group (*p* = 0.181). Delayed PPB occurred in 4 patients in the DOAC and 8 in the clopidogrel group (*p* > 0.999). No patient experienced a thromboembolic event in the 4 weeks after the polypectomy. Other patient characteristics are described in in Table 1.

**Table 1:** Characteristics of patients who underwent colonoscopic polypectomy.

Variables	DOAC group (n = 131)	Clopidogrel group (n = 270)	*p*-value
Age (year), mean ± SD	71.7 ± 9.01	67.5 ± 9.34	< 0.001
Male, n (%)	100 (76.3)	212 (78.5)	0.622
Comorbidities, n (%)			
Respiratory disease	16 (12.2)	12 (4.4)	0.004
Diabetes mellitus	37 (28.2)	88 (32.6)	0.378
Hypertension	86 (65.7)	177 (65.6)	0.985
Atrial fibrillation	121 (92.4)	14 (5.2)	< 0.001
Coronary arterial disease	25 (19.1)	122 (45.2)	< 0.001
Cerebrovascular disease	36 (27.5)	65 (24.1)	0.461
Chronic kidney disease	7 (5.34)	27 (10.0)	0.116
Antithrombotic discontinuation before procedure, day, mean ± SD	2.82 ± 1.74	5.84 ± 2.25	< 0.001
Compliance with antithrombotic discontinuation guideline, n (%)			0.181
Compliant	112 (85.5)	216 (80.0)	
Noncompliant (shorter discontinuation than guideline)	19 (14.5)	54 (20.0)	
Antithrombotic resumption after procedure, day, mean ± SD	1.65 ± 2.89	1.66 ± 1.06	0.989
Delayed PPB, n (%)	4 (3.1)	8 (3.0)	> 0.999
Thromboembolic event with 4 weeks of the procedure, n (%)	0	0	NA

*DOAC* Direct oral anticoagulant, *SD* standard deviation, *PPB* postpolypectomy bleeding, *NA* not applicable.

### Polyp and polypectomy procedure data

A total of 320 polyps in the DOAC and 669 polyps in the clopidogrel group were removed. The mean number of removed polyps in each patient was 2.46 ± 2.25 in the DOAC and 2.47 ± 2.55 in the clopidogrel group (*p* = 0.974). The average polyp size was 7.53 ± 4.00 mm in the DOAC and 7.71 ± 4.55 mm in the clopidogrel group (*p* = 0.548). Compared with the clopidogrel group, prophylactic hemostasis was more frequently performed in the DOAC group (22.3% versus 30.0%; *p* = 0.008). Immediate PPB occurred in 5.31% of the DOAC group and 6.58% of the clopidogrel group (*p* = 0.439). No difference in the delayed PPB rate was observed between DOAC and clopidogrel groups (1.6% in both groups; *p* = 0.924) (Table 2).

**Table 2:** Characteristics of the polyps and polypectomy procedure.

Variables	DOAC group (n = 320)	Clopidogrel group (n = 669)	*p*-value
No. of polyps per patient, mean ± SD	2.46 ± 2.25	2.47 ± 2.55	0.974
Polyp size, mm, mean ± SD	7.53 ± 4.00	7.71 ± 4.55	0.548
Location, n (%)			0.823
Right colon	198 (61.9)	409 (61.1)	
Left colon	122 (38.1)	260 (38.9)	
Polypectomy technique, n (%)			0.590
Cold snare polypectomy	86 (26.9)	193 (28.9)	
EMR	228 (71.3)	468 (70.0)	
ESD	6 (1.88)	8 (1.2)	
Histopathology, n (%)			0.831
Adenoma (LGD)	231 (72.2)	495 (74.0)	
Adenoma (HGD)	3 (0.9)	10 (1.5)	
Hyperplastic polyp	20 (6.3)	37 (5.5)	
Adenocarcinoma	10 (3.1)	13 (1.9)	
SSA/P	18 (5.6)	37 (5.5)	
Others	38 (11.9)	77 (11.5)	
Prophylactic clipping, n (%)	96 (30.0)	149 (22.3)	0.008
Immediate PPB, n (%)	17 (5.3)	44 (6.6)	0.439
Delayed PPB, n (%)	5 (1.6)	11 (1.6)	0.924

*DOAC* Direct oral anticoagulant, *EMR* endoscopic mucosal resection, *ESD* endoscopic submucosal dissection, *HGD* high-grade dysplasia, *LGD* low-grade dysplasia, *PPB* postpolypectomy bleeding, *SD* standard deviation.

### Risk factors for delayed PPB

Of 989 polypectomy sites, 16 developed delayed PPB (1.6%) and were successfully treated via endoscopic hemostasis (argon plasma coagulation for 1 and clipping for 15). According to the logistic regression analyses, the risk of delayed PPB increased in patients who showed immediate PPB relative to those who did not (odds ratio [OR] 5.218, 95% confidence interval [CI] 1.623–16.775, *p* = 0.006). The type of antithrombotic (DOAC vs clopidogrel) did not affect the risk of delayed PPB (OR 1.062, 95% CI 0.390–2.896, *p* = 0.906). Other patient-related and polyp-related factors showed no association with delayed PPB.

### Case-matched analysis

We conducted a propensity score matching to lower any effects of selection bias. Two hundred and seventy-six of the 320 polyps in the DOAC group were matched with the same number of polyps in the clopidogrel group as a control, based on age, sex, comorbidities (respiratory disease, diabetes mellitus, hypertension, and cardiovascular disease), polypectomy techniques (cold snare polypectomy vs. others), polyp size, location (right versus left colon), histopathology (adenoma vs. others), compliance with the antithrombotic discontinuation guideline, and prophylactic hemostasis (Table 3). Then, a logistic regression analysis was conducted, which revealed that DOAC did not increase the risk of delayed PPB in comparison with clopidogrel (OR 0.929, 95% CI 0.436 –1.975, *p* = 0.847).

**Table 3:** Characteristics of the 552 propensity score-matched cases.

Variables	DOAC group	Clopidogrel group	SMD
	Patient, n = 124	Patient, n = 145	
	Polyp, n = 276	Polyp, n = 276	
Age, year, mean ± SD	72.5 ± 8.6	72.3 ± 8.5	0.021
Sex			0.017
Male	208	210	
Female	68	66	
Comorbidities			
Respiratory disease	19	19	0.000
Diabetes mellitus	91	93	0.015
Hypertension	159	151	− 0.058
Cardiovascular disease	73	72	− 0.008
Polypectomy technique			0.017
Cold snare polypectomy	67	69	0.801
Others	209	207	
Polyp size, mm, mean ± SD	7.44 ± 3.87	7.74 ± 4.48	− 0.072
Location			0.030
Right colon	169	165	
Left colon	107	111	
Histopathology			− 0.040
Adenoma	209	201	
Others	67	75	
Compliance with antithrombotic discontinuation guideline	241	243	− 0.022
Prophylactic clipping	85	83	− 0.016

*DOAC* Direct oral anticoagulant, *SD* standard deviation, *SMD* Standardized mean difference.

## Discussion

In the current study, we evaluated the risk of delayed PPB and clinical outcome in patients taking DOACs or clopidogrel. Compliance rates to the antithrombotic discontinuation guidelines for polypectomy and delayed PPB rates were 85.5% and 3.1%, respectively, in the DOAC group and 80.0% and 3.0%, respectively, in the clopidogrel group. The propensity score-matched case–control analysis revealed no difference in the delayed PPB rate per polypectomy site between the two groups (1.6% in both groups). Meanwhile, a recent large retrospective study analyzed the risk of GI bleeding within 30 days after polypectomy in patients taking DOAC, clopidogrel, warfarin, or no antithrombotic agent^15^. It reported a delayed PPB rate in the DOAC group of 0.6%, and the risk of delayed PPB did not increase in the DOAC group in comparison with the control (no antithrombotic agent) group (OR 0.90, 95% CI 0.44–1.85). Conversely, the delayed PPB risk increased in the clopidogrel and warfarin groups in comparison with the control group (OR 2.84, 95% CI 2.16–3.73 in the clopidogrel group: OR 1.90, 95% CI 1.28–2.83 in the warfarin group). These results suggest that the risk of delayed PPB is smaller in those taking DOAC than in those taking clopidogrel or warfarin. A Japanese study recently reported the delayed bleeding rate of therapeutic endoscopic procedures (including polypectomy, endoscopic mucosal resection [EMR], and endoscopic submucosal dissection [ESD]) for lesions of the esophagus (2.1%), stomach (15.4%), duodenum (0.4%), and colon (82.1%)^16^. According to their predominantly colonic procedure data, the delayed bleeding rate was not significantly different between patients taking warfarin or DOAC (13.8% vs. 9.5%, respectively), but endoscopic hemostasis was more frequently required in those taking warfarin than DOAC (12.0 vs. 6.9%, p < 0.05). Therefore, taken together with the results of our study, the risk of delayed PPB between patients taking DOACs and those taking clopidogrel is deemed at least equivalent.

Unlike DOACs, the incidence of delayed PPB in patients taking clopidogrel is relatively well-known and ranges from 0.8 to 4% in previous reports^3,17,18^. In a meta-analysis including five observational studies, the pooled relative risk of delayed PPB was 4.66 (95% CI 2.37–9.17) if clopidogrel was not discontinued before polypectomy^6^. A randomized controlled trial (RCT) compared the PPB rate of those who continued thienopyridine (mostly clopidogrel) with that of controls (thienopyridine non-users). In that study, delayed PPB occurred in 2.4% of those who continued thienopyridine but none of the controls. In contrast, a recent double-blind RCT evaluated the effect of 7 days of clopidogrel interruption before polypectomy, which showed no significant difference in the delayed PPB rate between the uninterrupted clopidogrel (3.8%) and the placebo (3.6%) groups^19^. In the current study, delayed PPB occurred in 3.0% of patients in the clopidogrel group, comparable with the studies mentioned above.

We evaluated the potential risk factors of delayed PPB per polypectomy site and only immediate PPB was positively associated with the risk of delayed PPB. Therefore, if immediate PPB occurs in patients taking either DOAC or clopidogrel, these patients should be carefully monitored. A previous study suggested that EMR was an independent risk factor of delayed PPB^15^. However, no association of the polypectomy methods with the risk of delayed PPB was observed in our study, which may be due to the relatively small average polyp size in this study. In our study population, the DOACs included four major medicines: apixaban, dabigatran, edoxaban, and rivaroxaban. The pharmacological properties are different between each drug, and as such, the delayed PPB risk may be different among the various DOACs and may be influenced by the clinical setting. In a large population-based retrospective study regarding any kind of GI bleeding in patients taking DOACs, apixaban showed a relatively lower risk of GI bleeding than did dabigatran or rivaroxaban^20^. On the other hand, apixaban showed a higher risk of bleeding than rivaroxaban according to a Japanese multicenter retrospective study focused on GI bleeding after therapeutic endoscopy for patients taking DOACs^16^. Our data did not show any difference in delayed PPB according to the type of DOACs, which may be due to our limited number of cases.

This study has several limitations. Because of its retrospective design, the DOAC and clopidogrel groups differed with respect to the mean age of patients and the proportion of prophylactic hemostasis. One of the Korean reimbursement criteria for DOAC is nonvalvular AF in patients ≥ 75 years or with a history of a thromboembolic event, which may explain why the DOAC group was older than the clopidogrel group. Prophylactic hemostasis was also more often performed in the DOAC group than in the clopidogrel group, which may be because the endoscopists were less familiar with DOAC compared with clopidogrel. We used propensity score matching to perform a comparison between the DOAC and clopidogrel groups to overcome such differences. Meanwhile, our database includes a relatively small number of patients taking DOACs. Therefore, risk analysis was possible after propensity score matching for polyps in each group; however, risk analysis would not be appropriate after propensity score matching for patients in each group. Moreover, we could not examine the differences between the various DOACs in this study. Last, we did not compare the risk of delayed PPB in the antithrombotic-naive group with the DOAC or clopidogrel groups because a significant proportion of the retrospective data were not as qualified as the data available for the DOACs and clopidogrel groups. Therefore, prospective cohort studies should be followed to compare the risk of delayed PPB in patients taking antiplatelets, warfarin, and DOACs with those not taking such medicines. In order to provide critical evidence on the risk of PPB in those who taking DOACs further studies should be performed in the future, such as a RCT comparison between 2-day versus 1-day cessation of DOACs before polypectomy.

In conclusion, according to our retrospective, single-center study, the incidence of delayed PPB was 3.1% in patients taking DOACs, with the majority of patients following the antithrombotic discontinuation guidelines. The delayed PPB risk was not increased in patients taking DOACs in comparison with patients taking clopidogrel.

## Methods

### Patients

**Figure 1:**
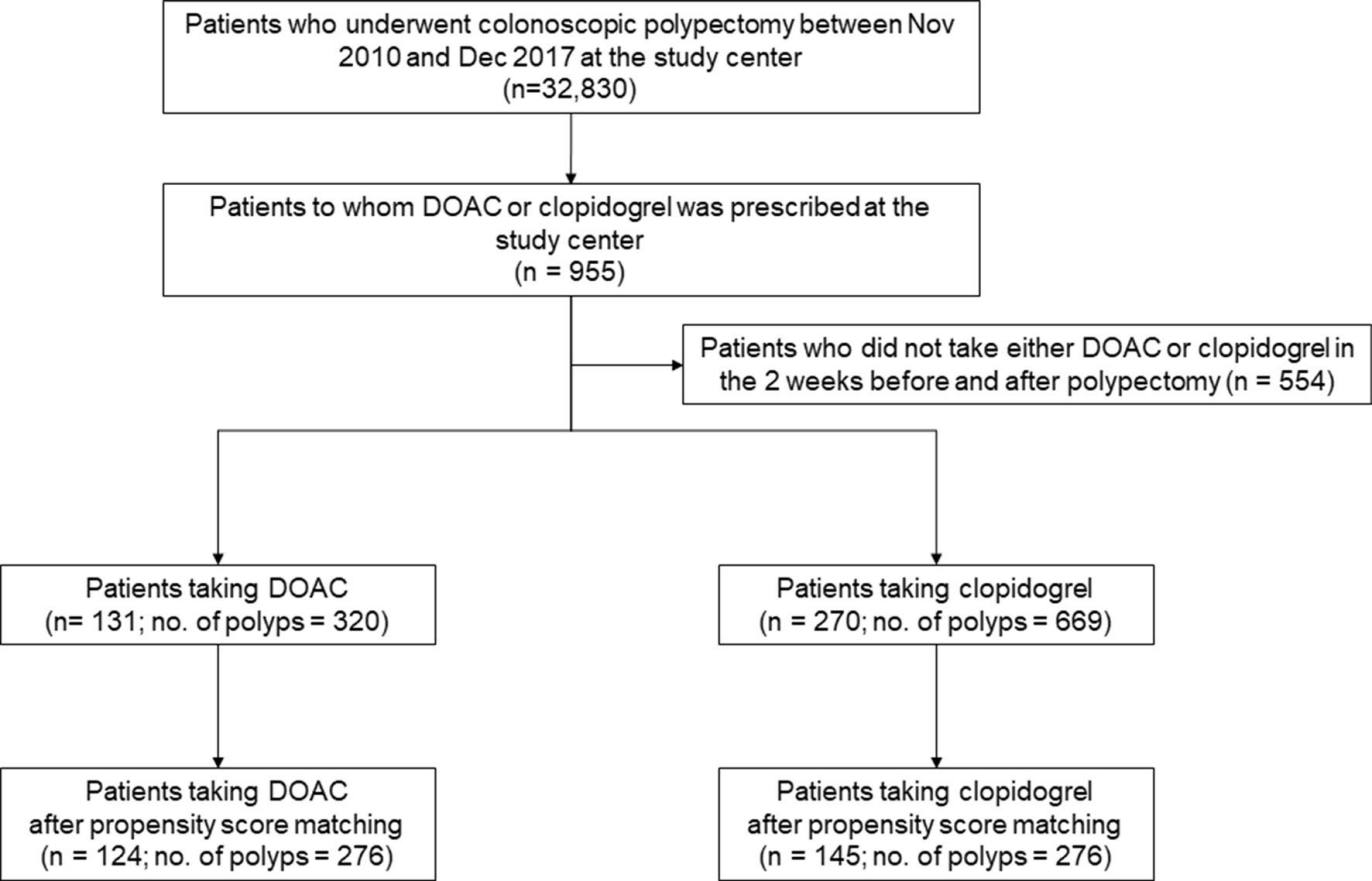
Flow chart for selecting patients taking either a direct oral anticoagulant or clopidogrel who underwent a colonoscopic polypectomy. *DOAC* Direct oral anticoagulant.

We investigated the retrospective data of patients taking DOACs and clopidogrel who underwent colonoscopic polypectomy by cold snare polypectomy, EMR, or ESD from November 2010 to December 2017 at a single referral center. Among 32,830 patients who underwent polypectomy during this period, 131 taking DOACs (DOAC group) and 270 taking clopidogrel (clopidogrel group) underwent colonoscopic polypectomy. All patients provided informed consent for the colonoscopic procedures. Patients taking DOAC or clopidogrel that was prescribed and/or monitored at the outpatient clinic of the same institution were included in this study. Patients who did not take either DOAC or clopidogrel in the 2 weeks before and after polypectomy were also excluded (Fig. 1). The clopidogrel group was included as a reference group. At 2–4 weeks after colonoscopic polypectomy, patients visited the outpatient clinic to check for adverse events and receive their pathologic results. During the consultation, a physician checked whether delayed PPB had occurred. We reviewed medical records for age, sex, medication before and after endoscopy, and comorbidities.

### Ethical approval

The study protocol followed the ethical guidelines of the Declaration of Helsinki Principles. The institutional review board of the Asan Medical Center approved this study (No. 2018–0184).

### Medication

We reviewed the data on medication for the use of clopidogrel and DOACs within 1 month preceding the colonoscopy. Data regarding the time intervals between drug cessation and the endoscopic procedure and drug resumption were retrieved. The DOAC group included patients taking apixaban, dabigatran, edoxaban, or rivaroxaban. The antithrombotic discontinuation guideline for polypectomy was considered to have been complied with if clopidogrel was interrupted 5 days before the procedure and DOAC 2 days before the procedure^4,5^.

### Endoscopic procedures

All procedures were performed using a single-channel colonoscope (CF-Q260AL/I, CF-H260AL/I, CF-HQ290AL/I; Olympus Medical, Tokyo, Japan). The endoscopic resection technique was determined by the endoscopists depending on the size and shape. Cold snare polypectomy was performed for lesions < 10 mm for which histology was estimated as benign. EMR was performed for nonpedunculated lesions between 10 to 20 mm in size or pedunculated lesions ≥ 20 mm. A 1:100,000 mixture of epinephrine saline solution was injected submucosally. After snaring the lesion, Endocut Q current (effect 2 or 3, cut duration 2, and cut interval 6) was applied via an electrosurgical unit (VIO300D; ERBE, Tubingen, Germany). ESD was performed according to our institutional protocol as described in previous studies^21–23^.

### Outcome measures and variables

We defined delayed PPB as the hematochezia that appeared after completion of the polypectomy, which required therapeutic interventions such as endoscopic hemostasis, angiography, and/or surgery. Immediate PPB was defined as intraprocedural hemorrhage that needed endoscopic hemostasis. In addition to the patients’ age, sex, and comorbidities, data regarding the time intervals between drug cessation, the endoscopic procedure, and drug resumption were retrieved. Characteristics of polyps and details of procedural outcomes were prospectively recorded in the structured histology reports and colonoscopy reports. Factors related to polyps and procedures were retrieved from the histology and colonoscopy reports. The location, size, and the final histology of the polypectomy specimen were investigated. The location of the polyp was classified to those on the right (from transverse colon to cecum) or the left colon (from rectum to splenic flexure). The procedure type, the occurrence of immediate PPB, and prophylactic hemostasis were also included as factors related to procedures. The methods and outcomes of hemostatic procedures were assessed in cases that had delayed PPB.

### Statistical analysis

The Student’s t-test and Pearson’s chi-square test were used to assess the differences between the two groups. Logistic regression analysis was performed to assess the risk factors for delayed PPB, including DOAC or clopidogrel use. Propensity scores were matched to minimize the potential bias due to unbalanced characteristics between the two groups and to improve causality between treatment and “delayed PPB” and “medication.” In calculating the propensity score, we considered factors that could influence outcomes including age, sex, drug discontinuation interval before polypectomy, execution of prophylactic hemostasis, location and size of the polyp, resection methods, and pathology of the resected specimen. The propensity score was calculated by the logistic model and matched between the two groups within a caliper of 0.07. For propensity score matching, AF and coronary artery disease were combined as cardiovascular disease. Resection technique was classified as cold snare polypectomy, and other techniques and histopathology findings were classified as adenoma and other findings, respectively. We also evaluated the balance between two groups using the standardized mean difference and Hosmer–Lemeshow test, and C-statistic was conducted to check the model fit. A *p*-value of < 0.05 was interpreted as statistically significant. All statistical analyses were performed using SAS software (version 9.4 m6 Cary, NC, USA).

## Data Availability

All data generated or analyzed during this study are included in this published article.
